# Characterization of zoonotic hepatitis E virus in domestic pigs and wild boar in Vietnam: Implications for public health

**DOI:** 10.1016/j.onehlt.2024.100857

**Published:** 2024-07-10

**Authors:** Le Chi Cao, Le Nguyen Nhat Ha, Tran Thi Giang, Vo Minh Tiep, Ngo Thi Minh Chau, Ton Nu Phuong Anh, Pham Khanh Duy, Le Phuc Nhan, Nguyen Thi Thu Hoai, Le Thi Kieu Linh, Nourhane Hafza, C. Thomas Bock, Truong Nhat My, Bui Tien Sy, Nguyen Linh Toan, Le Huu Song, Thirumalaisamy P. Velavan

**Affiliations:** a Institute of Tropical Medicine, University of Tübingen, 72074, Tübingen, Germany; b Department of Parasitology, Hue University of Medicine and Pharmacy (HUMP), Hue University, 49000 Hue, Viet Nam; c School of Biotechnology, International University, Vietnam National University Ho Chi Minh City, 70000 Ho Chi Minh City, Viet Nam.; d Research Center for Infectious Diseases, International University, Vietnam National University Ho Chi Minh City, 70000, Ho Chi Minh City, Viet Nam; e Division of Viral Gastroenteritis and Hepatitis Pathogens and Enteroviruses, Department of Infectious Diseases, Robert Koch Institute, 13353 Berlin, Germany; f Vietnamese-German Center for Medical Research (VG-CARE), 10000 Hanoi, Viet Nam; g 108 Military Central Hospital, 10000 Hanoi, Viet Nam; h Vietnam Military Medical University, 10000 Hanoi, Viet Nam; i Faculty of Medicine, Duy Tan University, 55000 Da Nang, Viet Nam

**Keywords:** Viral hepatitis E, Wildlife, Wild boar, Pig, One health, HEV genotypes 3, 4

## Abstract

Vietnam's unprecedented demand for meat from livestock, including pigs and farmed wildlife, underscores the importance of understanding zoonotic reservoirs for hepatitis E virus (HEV). This study aimed to identify and characterize circulating zoonotic HEV in domestic pigs and wild boar to understand genotype frequencies, transmission dynamics, and associated human health burdens. Rectal swabs, feces, and liver samples from 415 pigs and 102 wild boars were collected across various farms and slaughterhouses in central and southern Vietnam and screened for HEV RNA using nested PCR. HEV RNA-positive samples underwent sanger sequencing and genotyping. Overall, 10% (*n* = 54/517) of samples were HEV RNA-positive, with wild boars exhibiting the highest HEV positivity rate at 25%, followed by domestic pigs at 7%. Southern Vietnam showed a higher HEV RNA positivity rate (20%) compared to central Vietnam (7%). Notably, rectal swabs demonstrated the highest positivity rate (15%), followed by feces (8%) and liver (4%). HEV-3a was the predominant genotype at 85%, followed by HEV-4b at 9% and HEV-3f at 6%. While HEV-3a was distributed across both central and southern Vietnam, HEV-3f was exclusively detected in central Vietnam, and HEV-4b was identified in wild boar in southern Vietnam. These findings underscore the substantial prevalence of HEV in wild boars, emphasizing their potential as crucial zoonotic reservoirs alongside domestic pigs. Further investigations involving occupationally exposed individuals in high-prevalence areas are warranted to evaluate the human health impact of zoonotic hepatitis E and inform preventive measures. Regular epidemiological studies are imperative for assessing the prevalence and transmission of zoonotic HEV infections among common reservoirs, thereby aiding in the prevention of spillover events within the community.

## 1 Introduction

Hepatitis E virus (HEV) is a leading cause of acute viral hepatitis, particularly in low and middle-income countries, with limited access to basic sanitation and hygiene. Globally, an estimated 20 million infections and 3.3 million symptomatic cases of hepatitis E occur annually, resulting in around 56.600 deaths [1]. Hepatitis E typically resolves itself in certain population depending on the HEV genotype but poses a greater risk to high-risk groups in developing countries, such as pregnant women. In industrialized countries, organ transplant recipients, HIV-infected individuals and those with underlying liver disease also face significant risk regardless of the HEV genotype. Equally, the infections may lead to serious extrahepatic manifestations such as neurological sequelae, acute pancreatitis, kidney injury and thyroiditis [2].

HEV is a small single-stranded RNA virus with a genome length of approximately 7.2 kb. The virus exists in two forms: quasi-enveloped particles found in the blood of the infected hosts, and non-enveloped virions shed in the patient's stool. Belonging to the family *Hepeviridae* and the subfamily *Orthohepevirinae,* it comprises four genera: *Avihepevirus*, *Chirohepevirus*, *Rocahepevirus* and *Paslahepevirus*. Members of the *Paslahepevirus* genus (hepatitis E virus) are phylogenetically distinct and exhibit a broad host range, infecting humans, domestic and wild mammals [3]. Currently, eight HEV genotypes (HEV-1 to HEV-8) have been identified. Genotypes 1 and 2 exclusively infect humans, while genotypes 3, 4 and 7 infect both humans and animals, and genotypes 5, 6 and 8 solely infect animals [4].

Genotypes 1 and 2 are more prevalent in developing countries and primarily spread through the faecal-oral route, often causing waterborne HEV outbreaks, particularly in Africa and Asia [4]. Notably, the most recent HEV outbreak occurred in April 2023 in Wau, South Sudan, with a mortality rate of 5.5%, likely attributed by HEV-1 [5]. In contrast, infections with genotypes 3 and 4 primarily occur through zoonotic transmission, either via close contact with infected animals or by consuming contaminated food such as raw or undercooked meat [4]. These genotypes are common in developed countries and were initially thought to be imported solely through travel to endemic regions. However, the rising number of autochthonous human hepatitis E cases, sharing high similarity with swine HEV isolates, indicates evidence of zoonotic transmission in these regions [6].

HEV genotypes are associated with distinct clinical manifestations of the disease. Acute hepatitis typically results from infection with HEV-1 and HEV-2, whereas HEV-3, HEV-4, and HEV-7 can induce chronic hepatitis, particularly in immunocompromised patients [4]. Studies indicate variations in the pathogenicity of genotypes 3 and 4 [7,8]. Notably, HEV-3f is likely associated with higher viral loads and increased hospitalization rates compared to subgenotype 3c [7]. However, the clinical manifestations of HEV did not correlate with HEV-3 or its subtypes, as shown in patients with acute hepatitis E [9]. Additionally, patients infected with HEV-4 tend to exhibit higher alanine aminotransferase activity than those infected with HEV-3, potentially heightening the risk of fulminant hepatitis [8]. Over recent years, Southeast Asia and China have witnessed a surge in sporadic cases of HEV genotype (HEV-3 and HEV-4) [10,11], indicative of an emerging zoonotic HEV in these regions. Consequently, surveillance of zoonotic HEV infections in common reservoirs is imperative in these areas.

Since the initial identification of HEV strains in domestic pigs in the United States in 1997, these strains have been detected worldwide both in domestic and wild boar populations, displaying widely varying hosts [12,13]. Vietnam, renowned for its significant pig production and consumption, stands as a potential hotspot for swine hepatitis E virus. The first outbreak in Vietnam occurred in 1996 in the Southwestern region, suspected to have spread via the Hau River, although the genotype remain unidentified [14]. Since then, no further outbreaks of HEV have been reported. Several surveillance campaigns for HEV in animals and high-risk groups have been conducted in the Southern and Northern regions, revealing the circulation of genotypes 3 and 4 in domestic pigs [15,16]. Seroprevalence studies have reported HEV- IgG positivity ranging from 8% in pregnant women [11] to 27% in blood donors [17] and 53% in individuals exposed to pigs [18], highlighting a significant exposure in the Vietnamese population. Pigs comprised the largest proportion of livestock in Vietnam, accounting for 67% (23 million) in 2021 and 74% (25 million) in 2022 [19]. Additionally, around 217 farms in Vietnam raised approximately 7500 wild boars in 2021 [20], underscoring the importance of evaluating the risk of zoonotic disease transmission in this specific population.

Given the substantial burden of HEV infection in Vietnam, routine surveillance of the virus in the animal reservoir is imperative. This molecular epidemiological study aims to evaluate the distribution and genetic diversity of zoonotic HEV in domestic pigs and wild boars in Southern and Central Vietnam, regions.

## 2 Material and methods

### 2.1 Ethics statement

The study was approved by the ethics committee of Hue University of Medicine and Pharmacy, Hue University, Vietnam (H2022/020) and the animal ethics committee of the International University (IU) - Vietnam National University - Ho Chi Minh City (VNUHCM- August 2022).

### 2.2 Study design and sampling

**Fig. 1:**
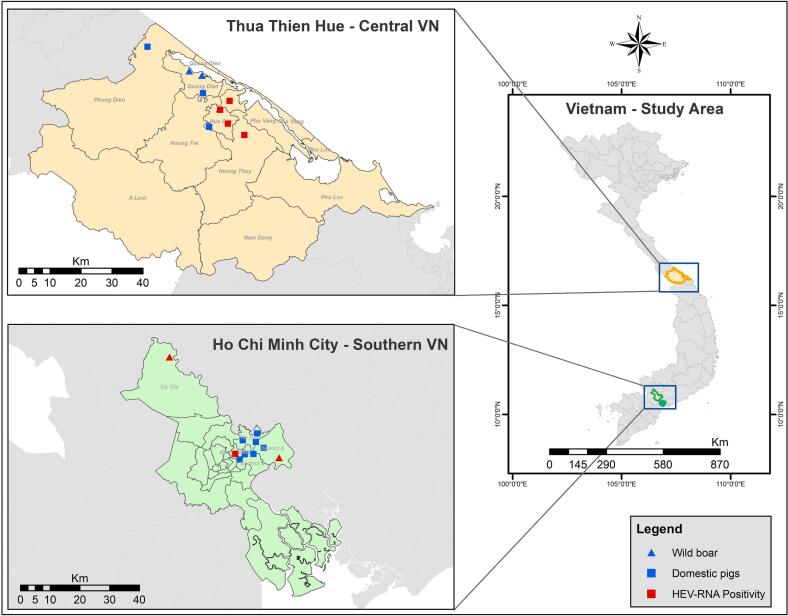
Zoonotic HEV screening from two study sites (Central and Southern Vietnam): The sampling sites for pigs are represented by squares, while the traingle indicate wild boar, which include slaughter houses, farms and wet markets. Sites where HEV positive samples were detected are highlighted in red. The map was created using ArcGIS 10.8 software.

From April to June 2022, 517 samples were collected from pigs and wild boars across Central and Southern Vietnam. In Thua Thien Hue province - Central Vietnam, liver samples (*n* = 199), rectal swabs (*n* = 92) and faecal samples (*n* = 77) were obtained from domestic pigs at seven study sites (six slaughterhouses and one farm). Additionally, two wild boar farms were sampled, resulting in six rectal swabs and eleven faecal samples were collected. In Ho Chi Minh City - Southern Vietnam, pig liver samples (*n* = 47) were collected from eight wet markets, while rectal swabs (*n* = 60) and faecal samples (*n* = 25) were obtained from wild boars from two different farms. Study locations are detailed in Fig. 1 and Table 1, and sampling adhered to standard operating procedures for One Health surveillance [21]. Liver tissue (approx. 1–2 g) was collected immediately post-slaughter, rectal swabs were individually labelled to prevent duplication, an. 2–3 faecal samples were collected from various locations within each pigpen. All samples were stored with DNA/RNA shield (Zymoresearch, Irvine, CA, USA), a solution that preserves genetic integrity, expression profiles and inactivates infectious agents at room temperature. The collected samples were then stored at -20 °C for subsequent analysis.

**Table 1:** HEV RNA positivity and genotypes distribution across sampling sites in Central and Southern Vietnam.

Region	Commune -District or City - Province	Source of samples #ID	Sample type	HEV RNA positivity	HEV RNA positivity (%); HEV genotypes
Central Vietnam (*n* = 385)	Phu Duong- Phu Vang-Thua Thien Hue	Slaughterhouse #1	Pig liver	0/21	(2%; n = 1/43); HEV3a
			Pig rectal swab	1/20	
			Pig feaces	0/2	
	Thuy Bieu-Hue city-Thua Thien Hue	Slaughterhouse #2	Pig liver	0/4	(0%; *n* = 14)
			Pig rectal swab	0/7	
			Pig feaces	0/3	
	Thuy Duong-Hue city-Thua Thien Hue	Slaughterhouse #3	Pig liver	2/126	(4%; n = 6/163) HEV3a; HEV3f,
			Pig rectal swab	2/18	
			Pig feaces	2/19	
	Thuy Chau-Huong Thuy-Thua Thien Hue	Slaughterhouse #4	Pig liver	6/48	(13%; n = 13/100); HEV3a
			Pig rectal swab	5/33	
			Pig feaces	2/19	
	Phong Hoa-Phong Dien-Thua Thien Hue	Slaughterhouse #5	Pig rectal swab	0/3	(0%; *n* = 04)
			Pig feaces	0/1	
	Phu Hau-Hue city-Thua Thien Hue	Slaughterhouse #6	Pig rectal swab	6/11	(26%; n = 8/31); HEV3a
			Pig feaces	2/20	
	Quang Tho-Quang Dien-Thua Thien Hue	Pig farm #1	Pig feaces	0/13	(0%; n = 13)
	Quang Loi-Quang Dien-Thua Thien Hue	Wild boar farm #1	Wild boar rectal swab	0/4	(0%; n = 13)
			Wild boar feces	0/9	
	Quang Loi-Quang Dien-Thua Thien Hue	Wild boar farm #2	Wild boar rectal swab	0/2	(0%; n = 04)
			Wild boar feces	0/2	
Southern Vietnam (*n* = 132)	An Phu-Cu Chi-Ho Chi Minh city	Wild boar farm #3	Wild boar rectal swab	13/25	(43%; n = 13/30); HEV3a
			Wild boar feces	0/5	
	Binh Trung Dong-District 9-Ho Chi Minh city	Wild boar farm #4	Wild boar rectal swab	6/35	(22%; n = 12/55) HEV3a; HEV4b
			Wild boar feces	6/20	
	Linh Trung-Thu Duc District-Ho Chi Minh city	Wet market #1	Pig liver	0/5	(0%; *n* = 05)
	Le Van Chi-Thu Duc-Ho Chi Minh city	Wet market #2	Pig liver	0/5	(0%; n = 05)
	Vo Van Ngan-Thu Duc-Ho Chi Minh city	Wet market #3	Pig liver	0/4	(0%; n = 04)
	Thao Dien-District 2-Ho Chi Minh city	Wet market #4	Pig liver	0/1	(0%; *n* = 01)
	Thao Dien-District 2-Ho Chi Minh city	Wet market #5	Pig liver	0/11	(0%; *n* = 11)
	Commune 1-Binh Thanh-Ho Chi Minh city	Wet market #6	Pig liver	1/8	(13%; n = 1/8) HEV3a
	Xo Viet Nghe Tinh- Binh Thanh-Ho Chi Minh city	Wet market #7	Pig liver	0/10	(0%; *n* = 10)
	Phuoc Long- District 9 —Ho Chi Minh city	Wet market #8	Pig liver	03	(0%; *n* = 03)

### 2.3 RNA extraction and cDNA synthesis

100 mg of liver tissue which was preserved in a DNA/RNA shield was washed twice with phosphate-buffered saline (PBS), that can help to stabilize the tissue and maintain the pH, creating an optimal environment for subsequent extraction procedures (Thermo Fisher Scientific, Frederick, USA) before RNA isolation. This step is essential to remove blood, extracellular proteins, and other contaminants that could interfere with the extraction process. Total RNA was extracted using TRIzol™ LS reagent (Thermo Fisher Scientific, Carlsbad, CA, USA) following the method described by Mendez et al. [22]. Samples were homogenised using a FastPrep 24™ homogeniser (MP Biomedicals, Santa Ana, CA, USA) by adding five glass beads to each tube (4–7 cycles of 30 s at 5 m/s) prior to isolate RNA according to the manufacturer's instructions. For the other sample types, 40 μl of rectal swab and 200 μl of faecal sample were resuspended in 100 μl PBS and 800 μl PBS respectively. A total of 140 μl of the rectal swab mixture was used for RNA isolation using QIAamp Viral RNA Kits (Qiagen GmbH, Hilden, Germany). 200 μl of the supernatant from faecal samples was used for RNA isolation after centrifugation at 10,000*g* for 2 min, using the same procedure as for the rectal swabs. The quality and quantity of 1 μg of extracted RNA were assessed using the Nanodrop (absorbance: 260/280 nm ratio) and the Qubit™ 4 fluorometer (Thermo Fisher Scientific, Waltham, MA, USA). The RNA was then transcribed into complementary DNA using the LunaScript RT SuperMix Kit (New England BioLabs, Ipswich, MA, USA).

### 2.4 Screening HEV using nested polymerase chain reaction (PCR)

All samples were tested for HEV RNA by nested PCR targeting the viral ORF1 and ORF2 regions as described by Hoan et al. [18]. For ORF1, the outer primer pairs were HEV-38 (sense) 5′- GAGGCYATGGTSGAGAARG-3′ and HEV-39 (antisense) 5′- GCCATGTTCCAGACRGTRTTCC-3′; while the inner primers were designated HEV-37 (sense) 5′- GGTTCCGYGCTATTGARAARG-3′ and HEV-27 (anti-sense) 5’-TCRCCAGAGTGYTTCTTCC-3′. For ORF2, the outer primers were HEV-34 (sense) 5’-CCGACGTCYGTYGAYATGAA-3′ and HEV-36 (anti-sense) 5’-TTRTCC TGCTGAGCRTTCTC-3′; inner primers were HEV-35 (sense) 5’-AAGTGAGCGCCTACAYTA YCG-3′ and HEV-29 (anti-sense) 5’-CTCGCCATTGGCTGAGAC-3′. The PCR amplification was performed in a 25 μL volume containing 50 ng viral cDNA, 1× PCR buffer, 0.4 mM dNTPs, 0.4 mM MgCl2, 0.6 μM specific primer pairs, and 1 unit of Taq polymerase (Qiagen GmbH, Hilden, Germany). The thermocycling parameters for the outer ORF1-PCR were an initial denaturation at 94 °C for 5 min, followed by 30 cycles of denaturation (95 °C for 30 s), annealing (56 °C for 30 s) and extension (72 °C for 30 s), followed by a final extension at 72 °C for 10 min. The thermocycling parameters for the inner ORF1-nested PCR were an initial denaturation at 94 °C for 5 min, followed by 36 cycles of denaturation (95 °C for 30 s), annealing (54 °C for 30 s) and extension (72 °C for 30 s), followed by a final extension at 72 °C for 10 min. For ORF2, the thermal cycling program was similar to ORF1, but the annealing temperature for PCR and nested PCR was 54 °C and 56 °C, respectively. A plasmid containing HEV cDNA served as a positive control. The amplicons (307 bp for ORF1/489 bp for ORF2) were visualized on 1.1% agarose gels stained with SYBR Green. Sample positive for ORF1 or ORF2 was considered positive for HEV RNA. All positive samples were replicated for confirmation.

### 2.5 HEV genotyping and phylogenetic analysis

PCR products were purified using the Exo-SAP-IT kit (USB, Affymetrix, Santa Clara, CA, USA) and utilized as templates for Sanger sequencing (Bigdye Terminator v3.1 Cycle Sequencing Kit; Applied Biosystems, Foster City, CA, USA) with the ABI 3130XL sequencing system. Sequence correction and alignment were conducted using DNASTAR-Lasergene v6 software (www.dnastar.com). Phylogenetic analysis of the ORF1 and ORF2 regions was carried out using MEGA 11 software (www.megasoftware.net) [23], employing the Maximum Likelihood method and the General Time Reversible (GTR) plus Gamma Distribution model. The statistical robustness and reliability of the branching order were confirmed via bootstrapping with 1000 replicates. The resulting phylogenetic tree was annotated and visualized using the online tool iTOL v6 (itol.embl.de) [24].

### 2.6 Data analysis

All analyses were conducted using GraphPad Prism (version 9.5.1). A *p*-value <0.05 was deemed statistically significant. Demographic data were depicted as mean values with corresponding ranges for quantitative variables, and as absolute numbers and percentages for categorical variables. Categorical data were assessed using Chi-square or Fisher's exact tests, while continuous variables were evaluated using *t*-tests or Kruskal-Wallis tests, as appropriate.

## 3 Results

### 3.1 Demographic and study population characteristics

This study was conducted in central and southern Vietnam, analyzing a total of 517 samples. In central Vietnam, 385 samples were collected from adult pigs and wild boars at six slaughterhouses, one pig farm, and two wild boar farms. The ages of the adult pigs and wild boars differed: pigs were 5–6 months old, while wild boars were 12–24 months old. In southern Vietnam, 132 samples were collected from adult pigs and wild boars at eight wet markets and two wild boar farms. The pigs had a body mass ranging from 60 to 100 kg. Sex determination was performed only on rectal swabs, with 98 samples from central Vietnam (34 females and 64 males) and 60 samples from southern Vietnam (33 females and 27 males). In southern Vietnam, 64% (85/132) of the samples were from wild boar population, including wild-boar piglets sampled between 24 and 160 days after birth (mean: 100 days ±54), with weights ranging from 4 to 35 kg each (mean: 17.2 kg ± 30.5).

### 3.2 HEV-RNA positivity

**Fig. 2:**
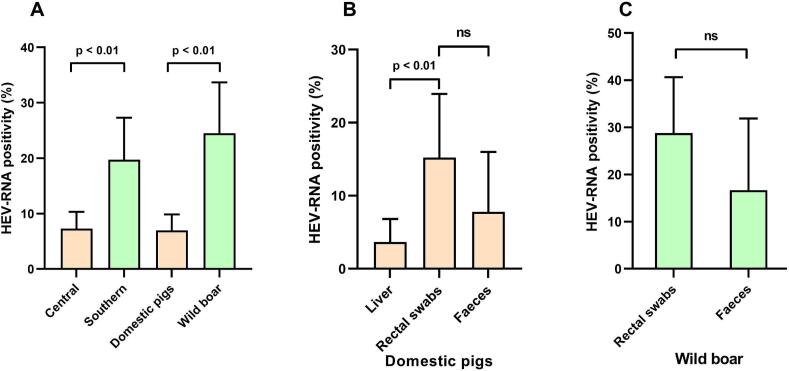
HEV positivity rates observed in this study: (A):Categorised by region and animals investigated; (B): Categorised by sample type in domestic pigs; (C): Cateogarised by sample type in wild boar; ns: non-significant

A total of 10% (*n* = 54/517) of samples were positive for HEV RNA with wild boars exhibited the highest HEV positivity rate at 25%, followed by domestic pigs at 7%. In central Vietnam, slaughterhouse Nr. 6 displayed the highest HEV positivity at 26% (*n* = 8/31), followed by slaughterhouse Nr. 4 at 13% (*n* = 13/100), slaughterhouse 3 at 4% (*n* = 6/163), and slaughterhouse Nr. 1 at 2% (*n* = 2/43). Similarly, in southern Vietnam, wild boar farm Nr. 3 showed the highest HEV positivity at 43% (n = 13/30), followed by wild boar farm Nr. 4 at 22% (*n* = 12/55) (Table 1). Additionally, a higher HEV RNA positivity rate was observed in southern Vietnam (20%) compared to central Vietnam (7%) (Fig. 2A). Among the sample types from domestic pigs, rectal swabs exhibited the highest positivity rate at 15%, followed by feces at 8%, and liver at 4%. Differences in RNA positivity were noted between rectal and liver samples. While wild boar rectal swabs showed a higher RNA positivity compared to feces (29% vs. 17%), this difference was not statistically significant (*p* = 0.17) (Fig. 2B and C).

### 3.3 Phylogenetic analysis

**Fig. 3:**
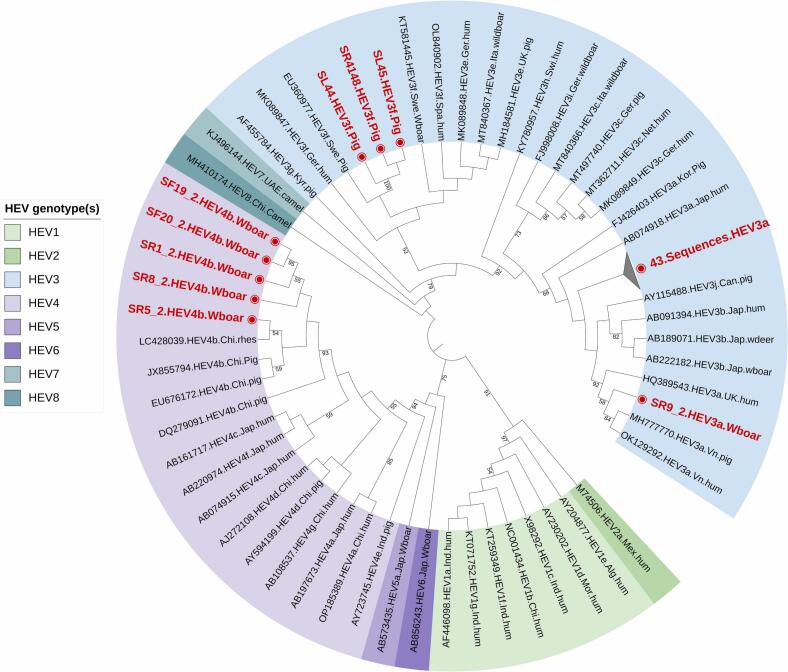
Phylogenetic analysis of HEV ORF-1 specific sequnces (n=52) obtained in this study from domestic pigs and wildboars. All positive (n=52) sequences are highlighted in red.

**Fig. 4:**
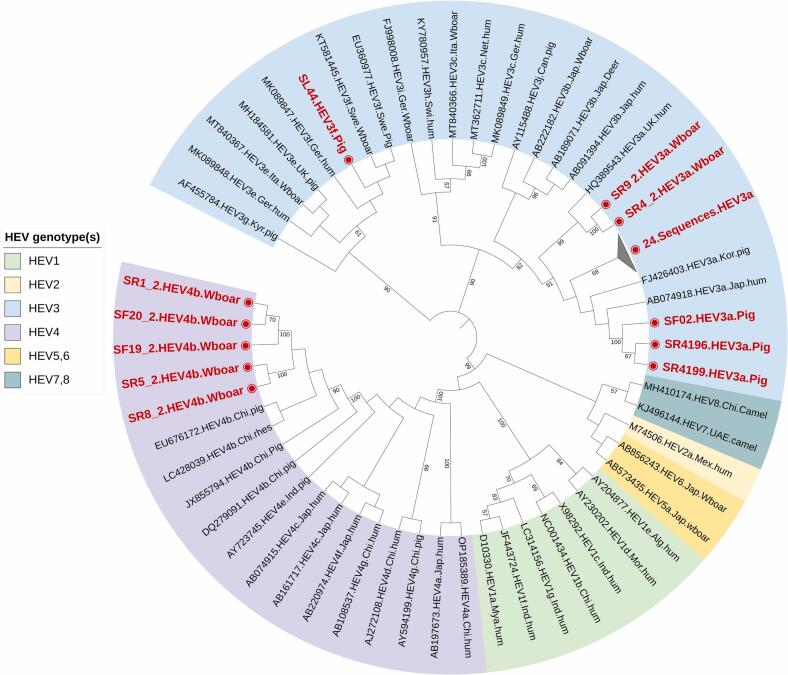
Phylogenetic anaylsis of HEV ORF-2 specific sequences (n=35) obtained in this study from domestic pigs and wild boars. All positive (n=35) sequences are highlighted in red.

The phylogenetic analysis of both HEV-ORF1 and ORF2 revealed HEV-3a as the predominant genotype at 85%, followed by HEV-4b at 9% and HEV-3f at 6% (Fig. 3, Fig. 4). HEV 3a genotype was found distributed across both central and southern Vietnam, regardless of the studied slaughterhouses, pig farms, wild boar farms, or wet markets in the region. However, HEV 3f was only detected in slaughterhouse Nr. 3 in central Vietnam, while HEV 4b was identified in wild boar farm Nr. 4 in southern Vietnam (Table 1). The nested PCR results for both HEV ORF-1 and ORF-2 were consistent, with samples positive for HEV ORF-1 also testing positive for HEV ORF-2. A total of 87 successfully sequenced samples were submitted to the NCBI GenBank database, with accession numbers for ORF1 ranging from PP504786 to PP504831 and PP150468 to PP150473 (*n* = 52), and for ORF2 ranging from PP531178 to PP531212 (*n* = 35).

## 4 Discussion

While zoonotic transmission of HEV and autochthonous HEV cases are well-documented in high-income countries, a growing body of research from Southeast Asia increasingly reports human HEV infections originating from animals. This trend suggests that the virus is emerging as a significant pathogen in the region [25,26]. In this study, the HEV RNA positivity rate of 10% aligns closely with rates observed in neighboring countries such as Laos (11.6%) [27], the Philippines (7.4%) [28], and Thailand (3%) [29]. Previous investigations in northern Vietnam revealed a 12% HEV positivity rate in pig livers [18], while southern Vietnam exhibited a higher rate of 19% [15], indicating geographical variation in HEV prevalence. Notably, no prior studies have explored the prevalence of HEV in the wild boar population in Vietnam. Wild boars are commonly hunted game animals, and HEV prevalence has been noted to be higher in European countries (8.7%) [30]. Germany, in particular, reported the highest prevalence with 56% RNA positivity in bile sample [30].

Our study reveals a high prevalence of HEV RNA positivity in wild boars, particularly notable in southern Vietnam at 29%, with wild boar farm Nr. 4 recording up to 43% positivity. These findings underscore the significance of wild boars as a reservoir for HEV alongside domestic pigs in Vietnam, highlighting the predominance of HEV-4 genotypes among the wild boar population. Despite wild boars often being raised for breeding alongside domestic pigs, which primarily carry HEV genotype 3, the cohabitation raises the potential for cross-transmission via the faecal-oral route. This scenario heightens the risk of HEV recombination, potentially leading to the emergence of new genotypes.

When comparing various sample types from the same animal, rectal swabs and faecal samples exhibited higher HEV positivity rates compared to liver samples, indicating their potential suitability for HEV monitoring in animals. A study in southern Vietnam reported a higher HEV RNA positivity in faecal samples (19%), than in rectal swabs (8.2%) [15]. Another systematic study on wild boar populations revealed the highest HEV positivity in bile (17%), followed by liver (10%), serum (7%), feces (5%) and meat (3%) [30]. HEV RNA is more frequently detected in stool samples than in liver samples due to several factors. HEV sheds into feces as non-enveloped virions, whereas in blood, it circulates in a quasi-enveloped form [31]. Although HEV primarily replicates in the liver and infects hepatocytes, a significant amount of the virus is excreted into bile and subsequently into the intestine [[32], [33], [34], [35]]. This results in a high concentration of the virus in stool, increasing the likelihood of RNA detection compared to liver samples, which may have lower and more localized viral quantities, thereby enhancing detection rates. Equally, liver biopsies or tissue samples may not capture all areas where the virus is present, potentially resulting in lower detection rates compared to stool samples, which are more uniform and likely to contain higher concentrations of the virus. This underscores that the choice of screening method using different sample types can significantly impact the observed prevalence of HEV infection in animals.

In our study, PCR primers targeting both ORF1 and ORF2 were utilized for detecting and characterizing HEV. The sensitivity for ORF1 was notably higher than for ORF2 (96% vs. 64%). This finding aligns with a comparative study by La Rosa et al., which demonstrated that nested PCR targeting ORF1 detects more HEV cases than ORF2 and ORF3 [36]. However, our study also revealed that while the ORF1 assay was effective for HEV identification at the genotype level, subtype characterization necessitated sequencing of ORF2 (capsid region). Notably, HEV3a was the most common subgenotype (85%), followed by HEV4b (9%) and HEV3f (6%). While HEV3a and HEV4b prevalence has been documented in pig populations in northern Vietnam [16,18], our study is the first to report the circulation of HEV4b in wild boar and HEV3f in domestic pigs in Vietnam. The emergence of the new subgenotype 3f in Vietnam suggests that the import and export of pigs may facilitate the movement of HEV strains and the introduction of new subtypes from Thailand and Cambodia. Farms in central Vietnam have imported breeding pigs from these regions, facilitating the spread of HEV subtypes. Genotypes 3 and 4 are widespread in pigs and wild boars globally, yet the distribution of subgenotypes varies regionally. In European countries, subgenotype 3e is predominant, followed by 3f and 3c, while in Asia, subtypes 3a, 3b, and 3f are prevalent, particularly in Indonesia, Thailand, Japan, and Vietnam [37]. Notably, genotype 4, previously limited to Asian countries, is now widespread across other continents [38].

The HEV3a sample from this study (#PP531208) showed 95% nucleotide identity with the HEV3a isolate (#OK129292) observed in a pregnant Vietnamese woman [11], suggesting potential zoonotic transmission. Furthermore, the growing evidence of acute HEV infection linked to the consumption of liver-containing sausages, as reported in Finland in 2024 [39], underscores the potential risk of HEV transmission in Vietnam. The increasing popularity of liver sausages in the Vietnam also raises concerns about foodborne HEV transmission if these products are not properly cooked. Our studies have primarily investigated HEV transmission in pig farms. However, pig farmers and slaughterhouse workers likely serve as reservoirs for HEV transmission within the community and contribute to spillover between the animal-human compartments. This hypothesis is supported by our previous findings, which indicated elevated IgG and IgM seroprevalence among individuals engaged in these occupations. Specifically, we identified significantly higher levels of anti-HEV IgG and anti- HEV IgM antibodies in pig farmers and slaughterhouse workers compared to pork meat vendor [18].

Growing evidence of genotype 4b infections in Vietnam, and Cambodia, indicate a potential emerging public health concern [40], while no clinical study has compared the severity of genotype 3 and 4 infections in hepatitis patients. A study by Schemmerer et al. suggested that infections with genotype 3e and 3f might be linked to more severe disease and higher mortality compared to other HEV3 subtypes [41]. The HEV 239 vaccine, known as Hecolin, was licensed in China in 2011, and gained authorization in Pakistan in 2020. It is also undergoing clinical studies in other countries, including India, Bangladesh**,** Nepal, and Indonesia. Despite there being four genotypes of HEV (HEV1–4), the vaccine, which is based on genotype 1 (ORF-2 capsid protein), is expected to offer cross-genotype protection against all four genotypes since they are characterized as one serotype in humans. A study evaluating the immunogenicity and safety of Hecolin in children is currently underway in South Africa through a placebo-controlled trial [42].

Our study had limitations: Firstly, unequal sample sizes of domestic and wild pigs in central and southern Vietnam hindered direct prevalence comparisons between regions. Secondly, the efficacy of our RNA extraction method could have been better assessed by incorporating internal or external controls, to provide a clearer evaluation of RNA recovery from liver tissues and stool samples. The HEV RNA recovery rates, as demonstrated in the study by Wang et al. [43], also ranged from 1.27% to 100%. Thirdly, due to the inability to collect serum samples, we were unable to determine HEV seroprevalence in animals. Lastly the lack of samples from occupationally exposed groups precluded an assessment of zoonotic transmission within the Vietnamese population.

## 5 Conclusion

Our study highlights the significant prevalence of HEV in wild boars in Vietnam, suggesting their potential role as an important zoonotic reservoir alongside domestic pigs. Further investigations involving occupationally exposed individuals in areas with high HEV prevalence are crucial to evaluate the human health impact of zoonotic hepatitis E. These epidemiological studies play a vital role in regularly assessing the prevalence and transmission of zoonotic HEV infections among common reservoirs, aiding in the prevention of spillover events within the community.

## Funding

This study was funded by the PAN-ASEAN Coalition for Epidemic and Outbreak Preparedness (PACE-UP; German Academic Exchange Service (DAAD) Project ID: 57592343), and the authors additionally thank the German Federal Ministry of Education and Research (BMBF; ID: BMBF01DP19006A) and the Vietnamese Ministry of Science and Technology (MOST; ID: NĐT/DE/21/07) for the HEPNET project.

## Institutional review board statement

The study was approved by the ethics committee of Hue University of Medicine and Pharmacy, Hue University, Vietnam (H2022/020), and the animal ethics committee of the International University (IU) - Vietnam National University - Ho Chi Minh City (VNUHCM- August 2022).

## CRediT authorship contribution statement

**Le Chi Cao:** Writing – original draft, Visualization, Validation, Methodology, Investigation, Formal analysis, Data curation. **Le Nguyen Nhat Ha:** Methodology, Investigation. **Tran Thi Giang:** Methodology, Investigation. **Vo Minh Tiep:** Methodology, Investigation. **Ngo Thi Minh Chau:** Project administration, Methodology. **Ton Nu Phuong Anh:** Project administration, Methodology. **Pham Khanh Duy:** Resources, Methodology. **Le Phuc Nhan:** Resources, Methodology. **Nguyen Thi Thu Hoai:** Supervision, Resources, Project administration. **Le Thi Kieu Linh:** Methodology, Investigation. **Nourhane Hafza:** Methodology, Investigation. **C. Thomas Bock:** Writing – review & editing, Supervision, Resources, Methodology. **Truong Nhat My:** Supervision, Project administration, Methodology, Investigation. **Bui Tien Sy:** Resources, Project administration, Methodology, Investigation, Funding acquisition. **Nguyen Linh Toan:** Supervision, Project administration, Methodology, Investigation. **Le Huu Song:** Supervision, Resources, Project administration, Methodology, Investigation, Funding acquisition. **Thirumalaisamy P. Velavan:** Writing – review & editing, Writing – original draft, Visualization, Validation, Supervision, Resources, Project administration, Methodology, Investigation, Funding acquisition, Conceptualization.

## Declaration of competing interest

The authors declare there are no competing interests. The funder has no role in the study design, data collection and analysis, decision to publish or preparation of the manuscript.

## Data Availability

All data generated or analysed during this study are included in this article. A total of 87 successfully sequenced samples were submitted to the NCBI GenBank database, with accession numbers for ORF1 ranging from PP504786 to PP504831 and PP150468 to PP150473 (n = 52), and for ORF2 ranging from PP531178 to PP531212 (n = 35).
